# Displacement of the Uterus

**Published:** 1859-02

**Authors:** J. G. Westmoreland

**Affiliations:** Prof. of Materia Medica and Medical Jurisprudence


### ARTICLE II.

*Displacement of the Uterus.* By J. G. WESTMORELAND, M. D., Prof. of Materia Medica and Medical Jurisprudence.

As the "ailments to whichflesh is heir to," most frequently met with, are those which more universally interest the votaries of the "healing art," we propose to offer a few thoughts on one of the difficulties met with as often at least as any other in the general practice of Medicine. The derangement to which we allude, is that which heads this article, and in its various forms, is the source of more vexation and trouble to the Physician, and suffering to the female patient, than almost any variety of disease with which we meet.

The term, "Displacement of the Uterus," does not presuppose organic disease of that organ, but an abnormal condition of those organs and tissues concerned in preserving the uterus in its proper position. Upon the round and broad ligaments and the vagina, does this viscus depend for its natural position.

But these supports fail to accomplish this object when certain unnatural conditions of the bladder and other surrounding viscera exist, while the uterus is unimpregnated, and of the abdominal parietes, when impregnated. Most of the causes operating directly upon the uterus, in the production of displacement, are purely mechanical. It is true, that irritation and other forms of local disease affecting the vagina and other parts connected with the womb, produce a state of relaxation sufficient to destroy their usefulness in maintaining it in its natural position; but under these circumstances the womb may remain itself comparatively healthy, and descend in the pelvis alone from the force of gravitation. More particularly, however, do we find retroversion and anteversion, to be the result of mechanical force in the form of distended bladder and bowels, while retaining its place in the pelvis, and by its own weight falling against the relaxed abdominal parietes, when in a gravid condition.

To this last form, particularly, is this article intended to call attention. Anteversion or an inclination forwards, of the impregnated uterus, is no doubt the source of more difficulty in parturition, than is generally supposed. When that relaxed condition of the tissues composing the abdominal walls in front exists, favorable to this deviation, the womb assumes a position unfavorable to the ready expulsion of its contents. The axis of the uterus does not correspond with the superior strait of the pelvis. Therefore the unavailing efforts of the woman are expended against the pelvis, instead of forcing the head of the child into the brim of the pelvis.

Cases of this kind sometimes occur, and are supposed to result from a disproportion of the size of the head to the capacity of the pelvis, and the woman allowed to suffer these ineffective throes for an indefinite time, or subjected to unnecessary and injurious interference per vaginam. The true condition may be determined under such circumstances by the lax condition and unnatural anterior projection of the abdomen, and may be readily corrected by a firm pressure of the hand against the abdomen just above the pelvis, during the uterine contraction.

Recently a case of this kind was placed under my care, in which, for eight or ten hours, the woman had undergone active pains, without engaging the head of the child in the bones of the pelvis. Pressure on the abdomen against the head of the child, as described above, resulted in the immediate advancement of the head, and the accomplishment of delivery in thirty minutes.

